# Weight management merits attention in women with infertility: a cross-sectional study on the association of anthropometric indices with hormonal imbalance in a Ghanaian population

**DOI:** 10.1186/s13104-019-4593-5

**Published:** 2019-08-28

**Authors:** William K. B. A. Owiredu, Peter Ntim Ofori, Cornelius Archer Turpin, Christian Obirikorang, Emmanuel Acheampong, Enoch Odame Anto, Eddie-Williams Owiredu, Evans Asamoah Adu

**Affiliations:** 1 Department of Molecular Medicine, School of Medicine and Dentistry, Kwame Nkrumah University of Science and Technology, Kumasi, Ghana; 2 Independent Development Research Consultant, Total Family Health Organization, Accra, Ghana; 3 Department of Obstetrics and Gynaecology, Komfo Anokye Teaching Hospital, Kumasi, Ghana; 4 School of Medical and Health Sciences, Edith Cowan University, Joondalup, WA Australia

**Keywords:** Anthropometric indices, Fertility hormones, Hormonal imbalance

## Abstract

**Objective:**

This study determined the association of anthropometric indices with hormonal imbalance among infertile women in a Ghanaian population.

**Results:**

Follicle stimulating hormone (FSH) levels (18.47 vs. 8.67, p-value = 0.002), and luteinizing hormone (LH) (12.43 vs. 8.01, p-value = 0.044) were higher in women with primary infertility compared with women presenting with secondary infertility. Waist circumference (WC) and waist-to-height ratio (WHtR) showed significant negative partial correlation with prolactin in both primary and secondary infertile women. Also a significant negative partial correlation was observed between BMI and prolactin in secondary infertile women only. Waist-to-hip ratio (WHR) showed a positive association with LH in both primary and secondary infertility. WHR also showed significant positive correlation to LH/FSH ratio in secondary infertility whereas body adiposity index (BAI) showed a negative correlation to LH/FSH ratio. In a correlation analysis of anthropometric measures with hormonal profile and causes of infertility as a fixed factor, the association between anthropometric indices and fertility hormones was largely dependent on the underlying causes of infertility.

## Introduction

Infertility is a recognised public health issue, which is associated with medical, psychosocial and economics burden according to the World Health Organization (WHO) [1, 2]. It is a problem of global shares, affecting an average of 8% to 12% of couples worldwide [2]. Countries within the Sub-Saharan Africa (SSA), southern and central part of Africa, records as high as 30.0% prevalence of infertility among couples [3–5]. In Ghana, the prevalence rate of infertility is reportedly 11.8% among women and 15.8% among men [6].

Overweight/obesity influences fertility by affecting ovulatory function [7]. Chang [8] indicated that the complex hormonal balance of the hypothalamic–pituitary–gonadal axis is affected by an individual’s body mass index (BMI). Thus, the interplay between fertility and obesity is the effect of obesity on menstrual disturbances and subfertility, although the exact mechanism has not been clearly elucidated [9, 10]. Pandey et al. [9] reported that overweight and obese women have poorer outcomes following fertility treatment. Thus, highlighting the significant effect of extreme weight on fertility especially among women.

Functional redundancy of the gonadotropin releasing hormone (GnRH) causes disruption of the normal secretion of luteinizing hormone (LH) and follicle stimulating hormone (FSH), which is involved in a number of reproductive disorders in women [11, 12]. Measurements of hormones including prolactin and thyroid stimulating hormones has a diagnostic value in the evaluation of women fertility function [13]. Seth et al. [10] in recent years reported an association between obesity indices and hormonal derangements among infertile women. Prolactin (PRL) levels, which is significant in evaluating female fertility, may be secreted from adipose tissues [14]. Thus, providing a link between obesity and hyperprolactinemia. In Ghana, studies evaluating the effect of weight-related behaviours on fertility hormones are lacking. Therefore, this study evaluated the association of anthropometric indices with hormonal imbalance among infertile women in a Ghanaian population.

## Main text

### Methods

The study was a cross-sectional study conducted at the Department of Obstetrics and Gynaecology at the Komfo Anokye Teaching Hospital. All patients visiting the department for infertility issues and above 18 years from September 2015, to March 2018 were included as a sample. Those who were psychologically, physically and socially fit after an investigation by a gynaecologist were selected to partake in the study. Women with infectious conditions such as human immunodeficiency virus (HIV), Hepatitis B and C, and Tuberculosis were excluded from the study. Patients on any kind of hormone treatment or those with LH and FSH levels suggestive of menopausal state were excluded from the study. In total, 184 infertile women were included.

A brief consultation was conducted and appointment made with participants based on knowledge of their last menstrual periods for blood samples to be taken for 21-day progesterone (ng/ml), LH (mIU/ml), FSH (mIU/ml) and PRL (ng/ml) levels. Participants were asked to complete a structured questionnaire which asked about age, and years of infertility. Causes of infertility were extracted from the patient’s folder. Weight (kg) of each participant was measured using a platform electronic scale. Waist and hip circumference were measured using non-extensible tape measure at the point of the umbilicus and the maximal gluteal position, respectively. A stadiometer was used for body height in a good standing posture. BMI was calculated as an index of body weight and height (kg/m^2^). WHR and WHtR were estimated from the ratio of waist (cm) to hip (cm) and waist (cm) to height (cm), respectively. Abdominal volume index (AVI) and BAI were calculated using the formulae by [15] and [16], respectively. Two millilitres fasting venous blood sample was drawn from the subject on the twenty-first day of menstrual cycle using standard venepuncture techniques into plain vacutainers without any additive. Serum separated after clotting was used for the estimation of fertility hormones using *mini* VIDAS^®^ Hormonal Analyser (BioMerieux^®^ SA, France).

Statistical analysis was done using Statistical package for Social Sciences (SPSS) version 25 for windows. Kolmogorov–Smirnov test was used to assess the normality of the data. Partial Pearson’s correlation analysis adjusted for age and duration of infertility were used to determining the correlation between anthropometric indices and hormonal factors. p-value < 0.05 was considered as statistically significant.

### Results

Women with secondary infertility were older compared with women with primary infertility (34.9 vs. 30.3 years, p-value < 0.0001). A higher proportions of women with primary infertility were young adults (79.6%) whereas a higher percentage of women with secondary infertility were middle aged adults (51.3%). Mean FSH (18.47 vs. 8.67, p-value = 0.002), and LH (12.43 vs. 8.01, p-value = 0.044) were higher for women with primary infertility compared with secondary infertility, respectively (Table 1).

**Table 1:** Basic characteristics of the study participants

Variable	Primary infertility (n = 108)	Secondary infertility (n = 76)	p-value
Duration of infertility^a^	4.0 ± 0.39	5.5 ± 0.42	*<* *0.0001*
Age (years)^a^	30.3 ± 0.56	34.9 ± 0.61	*<* *0.0001*
Age staging			
Young adults (19–34 years)	86 (79.6)	37 (48.7)	*<* *0.0001*
Middle-aged adults (≥ 35 years)	22 (20.4)	39 (51.3)	*<* *0.0001*
Aetiology			
Hyperprolactinemia	17 (15.7)	6 (7.9)	0.113
Tubal factors	30 (27.8)	8 (10.5)	*0.004*
Male factors	18 (16.7)	6 (7.9)	0.082
Ovulation problems	3 (2.8)	20 (26.3)	*<* *0.0001*
PCOS	16 (14.8)	15 (19.7)	0.380
Endometriosis	2 (1.9)	4 (5.3)	0.200
Uterine causes	14 (13.0)	14 (18.4)	0.310
Unexplained causes	14 (13.0)	11 (14.5)	0.769
Body mass index (kg/m^2^)^a^	28.6 ± 0.50	29.8 ± 0.54	0.116
Waist circumference (cm)^a^	88.6 ± 1.07	88.2 ± 1.07	0.832
Waist-to-hip ratio^a^	0.87 ± 0.01	0.86 ± 0.01	0.088
Waist-to-height ratio^a^	0.56 ± 0.01	0.55 ± 0.01	0.408
Body adiposity index^a^	32.3 ± 0.50	32.5 ± 0.64	0.816
Progesterone (ng/ml)^a^	8.94 ± 0.58	7.52 ± 0.58	0.547
FSH (mIU/ml)^a^	18.47 ± 2.18	8.67 ± 1.17	*0.002*
LH (mIU/ml)^a^	12.43 ± 1.25	8.01 ± 0.97	*0.044*
LH/FSH ratio^a^	1.00 ± 0.10	1.28 ± 0.18	0.061
Prolactin (ng/ml)^a^	20.41 ± 1.93	17.99 ± 1.58	0.060

All values are presented as frequency and column proportions, unless otherwise specified. Highlighted values are statistically significant

Values highlighted in italics are statistically significant

*PCOS* polycystic ovarian syndrome, *FSH* follicle stimulating hormone, *LH* luteinizing hormone

^a^Denotes values presented as mean ± standard error of mean

A significant negative partial correlation was observed between BMI and prolactin in secondary fertility (R = − 0.24, p-value = 0.036). WC had a negative association with prolactin in both primary (R = − 0.236) and secondary (R = − 0.232) infertility. WHtR had a significant negative partial correlation with prolactin in both primary (R = − 0.226) and secondary (R = − 0.256) infertility. WHR correlated positively with LH in both primary (R = 0.213) and secondary (R = 0.229) infertility. Also WHR showed significant positive partial correlation to LH/FSH ratio in secondary infertility (R = 0.299). BAI showed a negative partial correlation to LH/FSH ratio (R = − 0.263) (Table 2).

**Table 2:** Partial correlation of anthropometric measures with hormonal profile in primary and secondary fertility

Variable	Primary infertility					Secondary infertility				
	PRG	FSH	LH	PRL	Ratio	PRG	FSH	LH	PRL	LH/FSH
BMI										
R	0.029	− 0.028	0.025	− 0.09	0.046	0.09	0.123	0.204	*−* *0.24*	0.218
p-value	0.762	0.773	0.794	0.353	0.638	0.439	0.291	0.077	*0.036*	0.058
WC										
R	0.061	0.03	0.178	*−* *0.236*	0.085	0.169	− 0.039	0.013	*−* *0.232*	0
p-value	0.531	0.756	0.065	*0.014*	0.382	0.145	0.741	0.913	*0.044*	0.999
WHR										
R	0.118	0.049	*0.213*	− 0.098	0.149	0.089	− 0.062	*0.229*	− 0.151	*0.299*
p-value	0.222	0.616	*0.027*	0.311	0.124	0.443	0.594	*0.047*	0.192	*0.009*
WHtR										
R	0.027	− 0.021	0.116	*−* *0.226*	0.057	0.179	0.011	− 0.024	*−* *0.256*	− 0.103
p-value	0.783	0.832	0.232	*0.019*	0.56	0.122	0.924	0.838	*0.025*	0.377
BAI										
R	− 0.092	− 0.11	− 0.091	− 0.183	− 0.08	0.135	0.097	− 0.116	− 0.21	*−* *0.263*
p-value	0.342	0.258	0.351	0.058	0.408	0.244	0.405	0.317	0.068	*0.022*

Values highlighted in italics are statistically significant

*R* partial correlation coefficient (adjusted for age and duration of condition), *BMI* body mass index, *WC* waist circumference, *WHR* waist-to-hip ratio, *WHtR* waist-to-height ratio, *BAI* body adiposity index, *PRG* progesterone, *FSH* follicle stimulating hormone, *LH* luteinizing hormone, *PRL* prolactin

WC (R = 0.45) and WHtR (R = 0.38) had a significant partial positive correlation to LH among patients with polycystic ovarian syndrome (PCOS) as the cause of infertility. WC and WHtR had a significantly partial correlation to FSH (R = 0.64 and 0.58, respectively) among patients with hyperprolactinemia as the cause of infertility. Among patients with tubal cause of infertility, a significant partial negative correlation was observed between WC (R = − 0.53), WHtR (R = − 0.55), WHR (R = − 0.41), BAI (R = − 0.44) and prolactin. Also significant partial negative association was observed for WC, WHtR and BAI to FSH (R = − 0.35, − 0.33 and − 0.35, respectively) among patients with tubal causes of infertility. Moreover, among patients with tubal causes of infertility, WHR was positively correlated to progesterone (R = 0.40), whereas WHtR showed positive correlation to LH/FSH (R = 0.34). Among patents with male partner being the cause of infertility, BMI, WC, WHR and WHtR showed significant negative partial correlation to progesterone. Moreover, BMI showed negative partial correlation to LH/FSH ratio (R = − 0.53). Among patients with unexplained causes of infertility, WC, WHtR and WHR showed a significant partial correlation to prolactin (Table 3).

**Table 3:** Partial correlation of anthropometric measures with hormonal profile and aetiology of infertility as a fixed factor

Variables	Polycystic ovarian syndrome					Hyperprolactinemia				
	PRG	FSH	LH	PRL	Ratio	PRG	FSH	LH	PRL	LH/FSH
BMI										
R	0.15	0.09	0.08	− 0.07	0.14	0.11	0.40	0.33	− 0.35	0.22
p-value	0.433	0.646	0.667	0.714	0.465	0.637	0.065	0.134	0.111	0.321
WC										
R	0.18	0.31	*0.45*	− 0.15	− 0.04	0.33	*0.64*	0.37	− 0.40	0.27
p-value	0.335	0.100	*0.012*	0.436	0.822	0.133	*0.001*	0.089	0.067	0.222
WHR										
R	− 0.12	0.08	0.29	− 0.10	0.14	− 0.18	0.20	0.25	− 0.12	0.27
p-value	0.532	0.659	0.118	0.598	0.446	0.432	0.376	0.270	0.605	0.225
WHtR										
R	− 0.01	0.22	*0.38*	− 0.06	− 0.07	0.26	*0.58*	0.32	− 0.38	0.22
p-value	0.96	0.25	*0.040*	0.75	0.70	0.24	*0.010*	0.15	0.08	0.34
BAI										
R	− 0.06	0.12	0.22	− 0.02	− 0.15	0.30	0.40	0.14	− 0.26	0.04
p-value	0.755	0.523	0.238	0.911	0.44	0.182	0.067	0.539	0.240	0.847
	**Tubal cause**					**Ovulation cause**				
BMI										
R	0.30	− 0.24	− 0.17	− 0.25	− 0.01	0.08	0.01	0.00	− 0.20	0.09
p-value	0.068	0.160	0.327	0.130	0.957	0.731	0.951	0.985	0.372	0.692
WC										
R	0.27	*−* *0.35*	− 0.08	*−* *0.53*	0.31	0.13	− 0.05	− 0.02	− 0.33	0.15
p-value	0.113	*0.036*	0.655	*0.001*	0.060	0.559	0.821	0.915	0.140	0.515
WHR										
R	*0.40*	− 0.12	0.10	*−* *0.41*	0.28	− 0.33	− 0.25	− 0.13	− 0.03	0.33
p-value	*0.014*	0.481	0.551	*0.011*	0.100	0.140	0.254	0.555	0.908	0.134
WHtR										
R	0.22	*−* *0.33*	− 0.04	*− 0.55*	*0.34*	0.22	− 0.04	− 0.02	− 0.35	0.03
p-value	0.193	*0.049*	0.814	*<* *0.0001*	*0.043*	0.337	0.853	0.922	0.114	0.889
BAI										
R	− 0.10	*− 0.35*	− 0.13	*− 0.44*	0.23	*0.53*	0.25	0.19	− 0.41	− 0.23
p-value	0.541	*0.033*	0.445	*0.006*	0.173	*0.011*	0.268	0.402	0.057	0.312
	**Male factor**					**Unexplained cause**				
BMI										
R	*− 0.60*	0.27	− 0.04	− 0.33	*− 0.54*	− 0.06	− 0.06	0.08	0.20	0.28
p-value	*0.002*	0.218	0.842	0.119	*0.008*	0.785	0.79	0.719	0.36	0.183
WC										
R	*− 0.54*	0.27	0.35	0.08	0.23	− 0.06	− 0.26	− 0.09	*0.48*	0.35
p- value	*0.01*	0.22	0.11	0.73	0.291	0.787	0.218	0.692	*0.018*	0.098
WHR										
R	*− 0.50*	0.19	0.12	− 0.15	0.03	− 0.22	0.08	0.07	*0.49*	0.0
p-value	*0.02*	0.40	0.59	0.505	0.878	0.307	0.72	0.743	*0.014*	0.986
WHtR										
R	*− 0.56*	0.15	0.16	0.02	0.05	− 0.13	− 0.29	− 0.15	*0.49*	0.24
p-value	*0.010*	0.500	0.480	0.927	0.834	0.533	0.171	0.484	*0.016*	0.254
BAI										
R	− 0.21	− 0.04	− 0.06	0.05	− 0.13	− 0.04	*− 0.47*	− 0.31	0.20	0.26
p-value	0.330	0.867	0.772	0.813	0.554	0.872	*0.02*	0.142	0.347	0.219

Values highlighted in italics are statistically significant

*R* partial correlation coefficient (adjusted for primary and secondary infertility), *BMI* body mass index, *WC* waist circumference, *WHR* waist-to-hip ratio, *WHtR* waist-to-height ratio, *BAI* body adiposity index, *PRG* progesterone, *FSH* follicle stimulating hormone, *LH* luteinizing hormone, *PRL* prolactin

### Discussion

Our findings indicates that women with secondary infertility were older and had longer duration of infertility compared with those with primary infertility. This finding is consistent with the findings of Seth et al. [10] and highlights the independent role of age in fertility function of women [17]. This age phenomenon suggests decreased fecundity in secondary infertile women who have been successful in having their previous pregnancies. Ages beyond 32-years reflects decrease in egg quality in association with a gradual increase in circulating level of FSH, declines in circulating anti-müllerian hormone and inhibin B concentrations [18].

One important finding of this study was that fertility hormones including FSH and LH were higher and above normal range in women with primary infertility compared with secondary infertility. Additionally, although not significant, levels of progesterone and PRL were high in women with primary infertility compared with secondary infertility. This finding aligns the findings of Al-Turki [19]. Generally, FSH stimulates several follicles to mature and LH kindles ovulation by causing the dominant follicle to burst and release its eggs into the fallopian tube. High LH and FSH levels increase ovarian testosterone production, alter oestrogen production, and causes abnormalities with ovulation. The levels of LH and FSH, observed among primary infertile women is suggestive of a possible primary ovarian failure and poor pregnancy outcomes [18, 20].

Another significant finding in this study was the direct association observed between LH and the central adiposity index (WHR) in both primary and secondary infertile women. Besides, no association was observed between any adiposity index and FSH levels in both primary and secondary infertile women. This findings deviates from previous report, which reported a positive association between central adiposity indices and FSH levels but not LH [10]. De Pergola et al. [21] also reported an inverse of adiposity indices with LH and FSH levels, which deviates from our present finding. In our findings, central adiposity indices (WC and WHtR) were positively correlated with LH levels among PCOS associated infertility. In line with a previous study [22] women with PCOS are most likely to have central fat distribution, which is associated with hyperandrogenaemia. Also, a neuroendocrine mark of PCOS is persistently rapid GnRH pulsatility, which favours pituitary synthesis of LH over that of FSH and contributes to the increased LH concentrations [23].

This study also observed a negative association between central adiposity indices (WC and WHtR) and prolactin levels in both primary and secondary infertile women. This finding is incongruent with a previous finding [10], which rather observed a positive association. The unique finding of this study was that prolactin levels of women with tubal factors as the cause of infertility showed negative association with central adiposity indices (WC and WHtR). Women with unexplained causes of infertility showed positive association between WC, WHtR and prolactin levels, which suggest possible obesity-induce hyperprolactinemia and poor fertility outcomes. Thus, we speculated that the association between central adiposity and prolactin levels is influenced by the underlying causes of infertility which merits future investigations.

Among hyperprolactinemia-associated infertility, a positive association was found between FSH levels and markers of central adiposity (WC and WHtR). Hyperprolactinemia affects GnRH neurons and pituitary gland function to reduce secretion of LH and FSH, which represents an ovulatory disorder often associated with secondary amenorrhea or oligomenorrhea [24, 25]. Also, the interplay between fertility and obesity is the effect of obesity on menstrual disturbances [9, 10]. Thus, we hypothesised an investigative associations of serum measures of ovarian reserve and ultrasound measurements of antral follicle counts with body size especially in hyperprolactinemia-associated infertility, which could better expound this observation.

Our findings suggest a direct association between adiposity indices and various hormonal derangements, which is largely dependent on the aetiology of infertility. Intervention undertaken to control central and visceral obesity would definitely provide a beneficial effect by correcting the hormonal imbalance. Therefore, we recommend that an effective weight-management intervention among infertile women is beneficial for their hormonal milieu, more appropriate for fertility.

## Limitations

The lack of a control population or available anthropometric national data to compare our results is a major limitations of our study. Also, the measurements of thyroid stimulating hormones, glucose and lipids as well as oestrogen and testosterone would have been beneficial to clearly explain some of the findings between obesity and hormone levels, which is a limitation to this study.

## Data Availability

The datasets used and analyzed during the study are available from the corresponding author on reasonable request.

## Abbreviations

FSH: follicle stimulating hormone
LH: luteinizing hormone
PRL: prolactin
GnRH: gonadotropin releasing hormone
PCOS: polycystic ovarian syndrome
BMI: body mass index
WC: waist circumference
WHR: waist-to-hip ratio
WHtR: waist-to-height ratio
BAI: body adiposity index
